# National Association of Dental Examiners

**Published:** 1889-09

**Authors:** 


### Editorial.

### NATIONAL ASSOCIATION OF DENTAL EXAMINERS.

The National Association of Dental Examiners held its eighth annual session at Saratoga Springs, N. Y., commencing Tuesday, August 6, 1889.

The following State boards of examiners were represented: Colorado, P. T. Smith; Delaware, C. R. Jefferis, T. H. Gilpin; Georgia, A. G. Bouton; Illinois, C. R. E. Koch; Indiana, S. T. Kirk; Maryland, T. S. Waters; Massachusetts, L. D. Shepard, J. S. Hurlbut; New Jersey, F. A. Levy, J. G. Palmer; Ohio, J. Taft, H. A. Smith; Vermont, George H. Swift, James Lewis.

The following colleges were declared "reputable,"—that is, in the eyes of the association, as conforming to the standard which it has adopted and on which it will recognize their diplomas and admit their graduates to practice without requiring an examination:

American College of Dental Surgery, Chicago, Ill.

Baltimore College of Dental Surgery, Baltimore, Md.

Boston Dental College, Boston, Mass.

Chicago College of Dental Surgery, Chicago, Ill.

College of Dental Surgery, University of Denver.

College of Dentistry, Department of Medicine, University of Minnesota, Minneapolis, Minn.

Dental Department, Columbian University, Washington, D. C.

Dental Department of Northwestern University, Chicago, Ill.

Dental Department of Southern Medical College, Atlanta, Ga.

Dental Department of University of Tennessee, Nashville, Tenn.

Harvard University, Dental Department, Cambridge, Mass.

Indiana Dental College, Indianapolis, Ind.

Kansas City Dental College, Kansas City, Mo.

Louisville College of Dentistry, Louisville, Ky.

Minnesota Hospital College, Dental Department, Minneapolis, Minn. (Defunct.)

Missouri Dental College, St. Louis, Mo.

New York College of Dentistry, New York City.

Northwestern College of Dental Surgery, Chicago, Ill. (Defunct.)

Ohio College of Dental Surgery, Cincinnati, Ohio.

Pennsylvania College of Dental Surgery, Philadelphia, Pa.

Philadelphia Dental College, Philadelphia, Pa.

School of Dentistry of Meharry Medical Department of Central Tennessee College, Nashville, Tenn.

St. Paul Medical College, Dental Department, St. Paul, Minn. (Defunct.)
University of California, Dental Department, San Francisco, Cal.
University of Iowa, Dental Department, Iowa City, Iowa.
University of Maryland, Dental Department, Baltimore, Md.
University of Michigan, Dental Department, Ann Arbor, Mich.
University of Pennsylvania, Dental Department, Philadelphia, Pa.
Vanderbilt University, Dental Department, Nashville, Tenn.

Because a college is not on the list does not necessarily indicate that it is not a thoroughly reputable institution, as was shown in the case of the Dental Department of St. Louis College of Physicians and Surgeons. This institution applied for recognition, but owing to insufficient information, the fault of the college in not sending a representative, the association was unable to take final action.

Two new States—Delaware and Colorado—were admitted to membership. It seemed strange that the great State of Pennsylvania, with three colleges within her borders, representing fully one-third of the dental students in this country, should not feel sufficient interest in the enforcement of dental laws to send a delegate, but such was the case. We call upon the board to rise and explain its delinquencies in the matter. In view of the fact that there is some doubt existing in the minds of the profession regarding the constitutionality of laws governing the practice of dentistry and of medicine, and because of the recent decision of the Supreme Court of New Hampshire, declaring unconstitutional the law requiring a license as a requisite for practice in that State, it behooves the boards of the several States to support one another, and adopt such uniform action as not to trust the contest of the constitutionality of their laws in the courts, or they may find themselves without any law, which would be a serious retrogression.

Considerable other business was transacted. The standing Committee on Colleges, consisting of the following gentlemen, Drs. L. D. Shepard, C. R. E. Koch, C. R. Jefferis, F. A. Levy, and S. T. Kirk, was instructed hereafter to take cognizance of and investigate all charges against colleges, and report a revised list of colleges at each annual meeting; this same committee was also given authority to inquire into the proper equipment and organization of colleges not now on the list, in order to be able to report as to the capability of such institutions to give acceptable instruction, both as to the quality and quantity of its teaching.

The action of the officers in omitting the name of the University of Maryland, Dental Department, from the printed list of recognized colleges last year was sustained. A resolution intended to explain the term reputable was adopted, to the effect—

"That it is the sense of this association that no one should be permitted to assume the responsibilities of a dental practitioner until he shall have had at least three years' previous study and instruction, inclusive of three full terms of not less than five months each, in a properly organized and equipped dental college, provided that time spent in the study of medicine or graduation from a medical college may be credited on this requirement not to exceed the period of two years or two full terms of collegiate instruction; and recommending to such State boards of dental examiners as are by the laws of their respective States required to issue licenses to practise dentistry to all holders of diplomas from reputable dental colleges that they make such rules as shall require all colleges to make three full calendar years of study and the attendance upon three full college terms of not less than five months each a prerequisite to graduation; and that only such colleges as shall comply with this rule on or before the beginning of their scholastic year of 1890–91 should thereafter be considered as reputable; and that all State boards should, when their State laws permit the same, decline to grant a license to practice to any one who cannot produce evidence showing that he has spent at least three full years in study and preparation before attempting to assume the responsibilities of a dental practitioner."

The association showed every determination to stand by the National Association of Dental Faculties and assist it in enforcing its new departure, if necessary.

The following officers were elected: T. S. Waters, Baltimore, President; C. R. E. Koch, Chicago, Vice-President; F. A. Levy, Orange, N. J., Secretary-Treasurer.

Adjourned to meet at the time and place of the next meeting of the American Dental Association, at 9 A.M. of the first day.

